# Characteristics and metabolic potential of biliary microbiota in patients with giant common bile duct stones

**DOI:** 10.3389/fcimb.2023.1259761

**Published:** 2023-11-06

**Authors:** Chenguang Dai, Chunfang Xu, Lu Zheng, Min Wang, Zhining Fan, Jianxin Ye, Dongming Su

**Affiliations:** ^1^ Department of Pathology, Nanjing Medical University, Nanjing, China; ^2^ Department of Gastroenterology, First Affiliated Hospital of Soochow University, Suzhou, China; ^3^ Digestive Endoscopy Department, First Affiliated Hospital with Nanjing Medical University, Nanjing, China

**Keywords:** biliary microbial community, choledocholithiasis, 16S rRNA sequencing, metabolic function, bile acid

## Abstract

**Background:**

Endoscopic retrograde cholangiopancreatography (ERCP) is an effective minimally invasive operation for the management of choledocholithiasis, while successful extraction is hampered by large diameter of stones. Emerging studies have revealed the close correlation between biliary microbiota and common bile duct stones (CBDS). In this study, we aimed to investigate the community characteristics and metabolic functions of biliary microbiota in patients with giant CBDS.

**Methods:**

Eligible patients were prospectively enrolled in this study in First Affiliated Hospital of Soochow University from February 2022 to October 2022. Bile samples were collected through ERCP. The microbiota was analyzed using 16S rRNA sequencing. Metabolic functions were predicted by PICRUSTs 2.0 calculation based on MetaCyc database. Bile acids were tested and identified using ultra performance liquid chromatography-tandem mass spectrometry.

**Results:**

A total of 26 patients were successfully included into final analysis, 8 in giant stone (GS) group and 18 in control group. Distinct biliary microbial composition was identified in patients with giant CBDS, with a significantly higher abundance of Firmicutes at phylum level. The unique composition at genus level mainly consisted of Enterococcus, Citrobacter, Lactobacillus, Pyramidobacter, Bifidobacterium and Shewanella. Pyramidobacter was exclusively found in GS group, along with the absence of Robinsoniella and Coprococcus. The contents of free bile acids were significantly higher in GS group, including cholic acid (98.39μmol/mL vs. 26.15μmol/mL, *p*=0.035), chenodesoxycholic acid (54.69μmol/mL vs. 5.86μmol/mL, *p*=0.022) and ursodeoxycholic acid (2.70μmol/mL vs. 0.17μmol/mL, *p*=0.047). Decreasing tendency of conjugated bile acids were also observed. Metabolic pathways concerning cholelithiasis were abundant in GS group, including geranylgeranyl diphosphate biosynthesis, gluconeogenesis, glycolysis and L-methionine biosynthesis.

**Conclusions:**

This study demonstrated the community structure and metabolic potential of biliary microbiota in patients with giant CBDS. The unique biliary microbial composition holds valuable predictive potential for clinical conditions. These findings provide new insights into the etiology of giant CBDS from the perspective of biliary microbiota.

## Introduction

Choledocholithiasis is a prevalent issue on a global scale. Common bile duct stones (CBDS) usually result from the migration of gallstones and exhibit a prevalence of 10-20% among individuals with symptomatic gallstones (Williams et al., 2017). Endoscopic retrograde cholangiopancreatography (ERCP) serves as an effective strategy for treating CBDS, often achieving a high rate of duct clearance (Committee et al., 2019). The successful extraction is hampered by a variety of reasons, such as large size of stone (Williams et al., 2017; Committee et al., 2019; Manes et al., 2019). Commonly, giant CBDS are more difficultly to be removed from duodenal papilla.

Microbiota is now recognized as a unique system in human body. With the development of sequencing technology, a lot of studies confirmed the existence of microbiota in bile (Nicoletti et al., 2020), even though biliary duct was considered as a sterile system. Biliary microbial clusters were found from various individuals, including healthy donors (Molinero et al., 2019) and several hepatobiliary diseases (Nicoletti et al., 2020). Different richness, diversity and composition of microbial community were revealed, especially CBDS (Wu et al., 2013; Tan et al., 2022). Previous studies concerning CBDS have often focused on the biliary microbial niches in recurrent CBDS (Shen et al., 2020; Li et al., 2022; Tan et al., 2022) or sphincter of Oddi disorders (Liang et al., 2016; Zhang et al., 2020). Until now, no study has been reported to describe the characteristics of biliary microbiota in patients with giant CBDS. It is essential for enhancing the treatment of giant CBDS to understand the features of biliary microbiota.

In the present study, we used 16S rRNA sequencing technology to reveal the microbial community within bile samples extracted from patients with giant CBDS. Metabolic functions were predicted through bioinformatic analysis at the same time. Afterwards, we applied ultra performance liquid chromatography-tandem mass spectrometry (UPLC-MS/MS) system to test the bile samples and identify the 15 bile acids quantitatively.

## Methods

### Study design and patient enrollment

The present study was designed as a case-control study. Consecutive patients were prospectively enrolled from February 2022 to October 2022 in First Affiliated Hospital of Soochow University. The inclusion criteria were as follows: (1) CBDS were confirmed by abdominal ultrasound, computed tomography (CT), magnetic resonance cholangiopancreatography (MRCP) or endoscopic ultrasound (EUS); (2) absence of antibiotics usage within the preceding three months. The exclusion criteria were as follows: (1) accompanied with acute obstructive suppurative cholangitis; (2) a history of surgery involved in upper gastrointestinal tract or common bile duct (CBD); (3) a history of gastrointestinal or hepatobiliary-pancreatic neoplastic diseases. All the patients eligible for this study provided with written informed consents, and they were allowed to drop out for any reason. In this study, the CBDS was categorized as a “giant stone” if its diameter was larger than 15mm, and the diameter ratio of the stone to the CBD exceeded 1.0. Based on the definition, the patients were assigned into two groups, giant stone group (GS group) or control group (not meeting the special criteria).

### Sample collection

ERCP was performed on the patients as soon as possible when informed consents were obtained. Duodenoscope was strictly disinfected before operation to keep the working channel sterile. All the instruments were sterile as well, including guide wire, aspiration catheter and the tube for bile storage. During the standard ERCP procedure, guide wire was firstly inserted into CBD under X-ray. Before the injection of contrast medium, bile samples were aspired through a sterile catheter. 5-10ml of bile was collected from each patient. And then the samples were immediately put into -80°C freezer for storage.

### DNA extraction, PCR amplification and 16S rRNA sequencing

Microbial DNA was extracted from bile samples using the QIAamp^®^ DNA Mini Kit (250) (QIAGEN, Germany) according to manufacturer’s protocols. The mixture containing InhibitEX Buffer and bile sample was homogenized and beat with 60 Hz for 1 min twice with a Homogeneous instrument (FASTPREP-24, Aosheng Biotech, China). Total DNA quality was examined by Thermo Nano Drop 2000 UV microspectrophotometer and 1% agarose gel electrophoresis. The details about DNA quality control were showed in **Supplementary Table 1**.

The V3-V4 region of the microbial 16S rRNA genes were amplified by PCR (95°C for 3min, followed by 30 cycles at 98°C for 20s, 58°C for 15s, 72°C for 40s and a final extension at 72°C for 5min) using primers 341F 5’-CCTACGGGRSGCAGCAG-3’ and 806R 5’-GGACTACVVGGGTATCTAATC-3’ (Wang and Qian, 2009). PCR reactions were performed in 25μL mixture containing 12.5μL of KFX HiFi 2×PCR Master Mix, 1μL of each primer (10μM), 50ng of template DNA and ddH2O. Amplicons were extracted from 2% agarose gels and purified using the AxyPrep DNA Gel Extraction Kit (Axygen Biosciences, Union City, CA, U.S.). The products were quantified using Qubit^®^2.0 (Invitrogen, U.S.). Based on the preparation of library, purified amplicons were submitted to 250bp paired-end sequencing on Illumina NovaSeq platform (Illumina, Inc., CA, USA).

### UPLC–MS/MS analysis of bile acids

An aliquot of 50μL bile sample was spiked with 100μL isotope internal standard and 50μL methanol, and then vortexed for 10min to precipitate the proteins. The mixture was centrifuged at 14,000rpm for 10min. Afterwards, 50μL supernatant was diluted and vortexed for mixing. Finally, 20μL sample was injected into the UPLC-MS/MS system (Amplatz et al., 2017; Yin et al., 2017).

The AB SCIEX Triple Quad 6500+LC/MS/MS system was used to identify the 15 bile acids. The ion source was an electric spray ion source in negative ion mode. The ion source parameters were as follows: 60 psi for desolvent gas, 55 psi for heating gas, 500° C for desolvent gas, 30 psi for Curtain Gas, 12 psi for Collision Gas and - 4500 V for spray voltage. The Analyst 1.6.3 software and MultiQuant software were used for data acquisition and quantitative analysis. The scanning mode was multiple reaction monitoring mode. The chromatographic separation was performed on a ZORBAX Eclipse XDB-C18 column (4.6*150mm 5μm, Agilent). The mobile phase consisted of 5mM ammonium acetate and methanol. The duration of gradient elution was 9 minutes.

Before acquiring the data, several blanks were injected firstly to balance the instrument. The injection sequences were blanks, calibration curves, blanks, quality control (QC) samples, samples and QC samples. The accuracy range of the 15 bile acids were 84.04%~119.97% in QC samples (**Supplementary Table 2**). The QC accuracy was with ±20% of target value.

### Data analysis

Demographic characteristics were recorded as detailed as possible, including age, sex, body-mass index (BMI), stone number, laboratory tests and past medical history. Statistical analysis was performed using SPSS statistics 27.0. Continuous variables, including bile acids, were described as mean with standard deviation (mean ± SD) or median and tested using Student’s t test or Mann-Whitney test. Categorical variables were described as count and percentage and compared using Chi-square test or Fisher’s exact test. *p*<0.05 was considered statistically significant. Correlation analysis between every two variables was carried out using spearman method.

When the bile samples were successfully sequenced, tags, trimmed of barcodes and primers were further checked and filtered according to the quality. 16S tags would be screened out if they were shorter than 250 bp or longer than 500 bp. Bases achieving a Phred score of over 30 (Q30) and containing fewer than 1 ambiguous N were retained. After enumerating the copy number of tags, redundancy of repeated tags was removed. Only the sequences with frequency greater than 1 were clustered into Operational Taxonomic Units (OTUs). OTUs were clustered on a 97% similarity threshold using UPARSE (http://drive5.com/uparse/), and chimeric sequences were analyzed and removed by Userach (version 7.0). The taxonomy of each representative sequence was identified by RDP Classifer (http://rdp.cme.msu.edu/) against the RDP database (http://rdp.cme.msu.edu/) with a confidence threshold of 80%.

OTU profiling table and alpha/beta diversity analyses were computed by QIIME (version 1.9.1). Sequencing depth was assessed by goods coverage index. Community richness and diversity were evaluated by Chao1, observed species, Shannon and Simpson indexes. Principal coordinates analysis (PCoA), nonmetric multidimensional scaling (NMDS) and heatmap were employed to assess microbial composition and distribution between the two groups based on weighted UniFrac distance, with significance determined through Adonis test. Core microbiome (Venn diagram) was drawn by R/Perl SVG. Linear discriminant analysis (LDA) Effect Size (LEfSe) was utilized to estimate the impact of each component abundance. The LDA threshold was set at 2.0. Wilcoxon analysis was introduced to find the distinct bacteria between the two groups, and *p*<0.05 was considered statistically significant. Both the analyses aimed to identify the genera that have a significant differential effect on sample division. Spearman correlation heatmap depicting relationships between genera was figured out based on the top 30 different genera using corrplot package in R.

Metabolic pathways were calculated by PICRUSTs 2.0 based on MetaCyc database to predict the metabolic functions. Principal Component Analysis (PCA) was used to illustrate the different distribution of microbiota and metabolic functions between the two groups. To reveal the relationship between differential genera and bile acids, the correlation was calculated by cor.test in R. And the heatmap was drew by heatmap.2 package.

## Results

### Patient demographics and preoperative characteristics

Totally 32 patients were eligible for the study, 8 in GS group and 24 in control group. 6/24 samples in control group were not successfully constructed the library, while all the samples in GS group were successfully constructed. As a result, we included 26 patients into statistical analysis, 8 in GS group and 18 in control group.

Patients in GS group was significantly older than control group. Patients in the two groups were similar in gender, BMI and coexisting disorders (**Table 1**). More than half the patients had multiple stones (GS group *vs.* control group, 75.0% *vs.* 55.6%, *p*=0.420) (**Table 1**). 7 patients in GS group and 10 patients in control group suffered from cholecystolithiasis at the same time (GS group *vs.* control group, 87.5% *vs.* 55.6%, *p*=0.095) (**Table 1**). Preoperative laboratory tests were comparable between the two groups, expect for serum albumin (GS group *vs.* control group, 32.11 ± 6.72g *vs.* 38.44 ± 5.76g, *p*=0.022) (**Table 1**).

**Table 1:** Patient demographics and preoperative characteristics.

	GS group n=8	Control group n=18	*p* value
Age, years (mean ± SD)	83.50 ± 6.28	66.67 ± 14.48	<0.001
Gender, male/female	6/2	13/5	1.000^§^
BMI^†^, kg/m^2^ (mean ± SD)	20.43 ± 3.06	22.54 ± 2.11	0.052
Stone no., single/multiple	2/6	8/10	0.420^§^
Past medical history, no. (%)			
Hypertension	2 (25.0%)	6 (33.3%)	0.667^#^
Diabetes	0 (0)	7 (38.9%)	0.062^§^
Gallstone	7 (87.5%)	10 (55.6%)	0.095^#^
Others^‡^	6 (75.0%)	14 (77.8%)	1.000^§^
Hb, g/L, (mean ± SD)	120.14 ± 24.51	133.88 ± 17.96	0.146
WBC, *10^9^/L, (mean ± SD)	6.13 ± 2.74	6.79 ± 3.15	0.638
PLT, *10^9^/L, (mean ± SD)	170.86 ± 107.30	184.88 ± 64.71	0.700
TBIL, μmol/L, (median)	24.55	20.10	0.429
DBIL, μmol/L, (median)	17.10	8.80	0.198
ALT, U/L, (median)	73.15	99.00	0.935
AST, U/L, (median)	51.10	35.70	0.397
γ-GGT, U/L, (median)	444.80	255.00	0.574
ALP, U/L, (median)	276.20	123.65	0.110
ALB, g, (mean ± SD)	32.11 ± 6.72	38.44 ± 5.76	0.022
CA19-9, U/mL, (median)	15.47	9.22	0.261

^§^Fisher’s exact test.

^#^Likelihood ratio.

^†^BMI: Body-mass index.

^‡^Other coexisting disorders included cerebral infarction, Parkinson’s disease, lung cancer, bronchiectasis, gout, hepatitis B, liver cirrhosis, coronary heart disease and atrial fibrillation.

### Distinct biliary microbial composition in patients with giant CBDS

The average sequence length was between 420bp and 440bp (**Supplementary Figure 1A**). The goods coverage index, a measure of sequencing depth, was comparable between the two groups (*p*=0.230), and both approached to 1.00 (**Supplementary Figure 1B**, **Supplementary Table 3**). These findings underscore the adequacy of the sequencing depth in this study.

**Figure 1:**
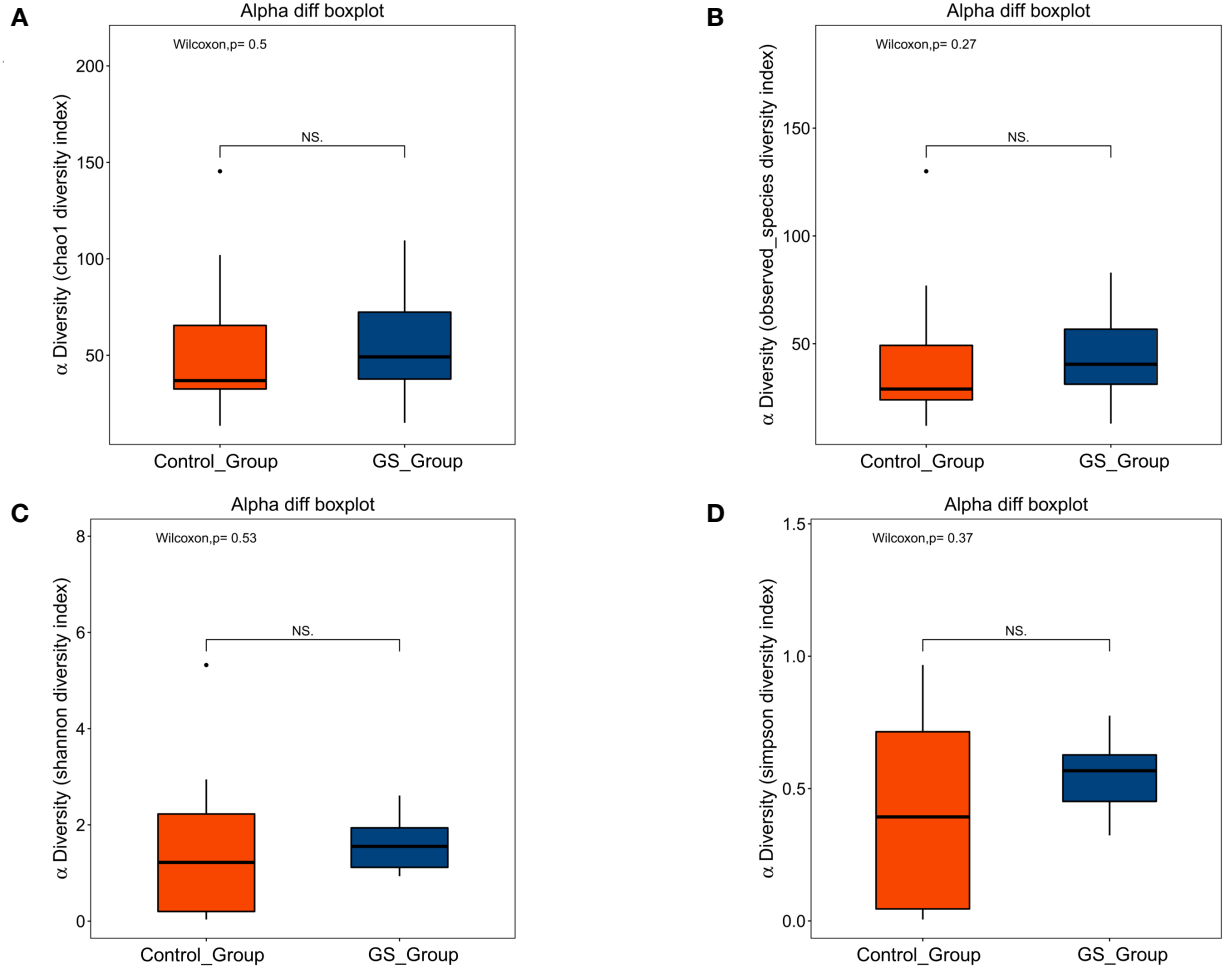
Alpha diversity analysis showed no significant difference in richness of biliary microbiota between the two groups: **(A)** Chao1 index (*p*=0.495); **(B)** Observed species index (*p*=0.266); **(C)** Shannon index (*p*=0.531); **(D)** Simpson index (*p*=0.367).

**Figure 2:**
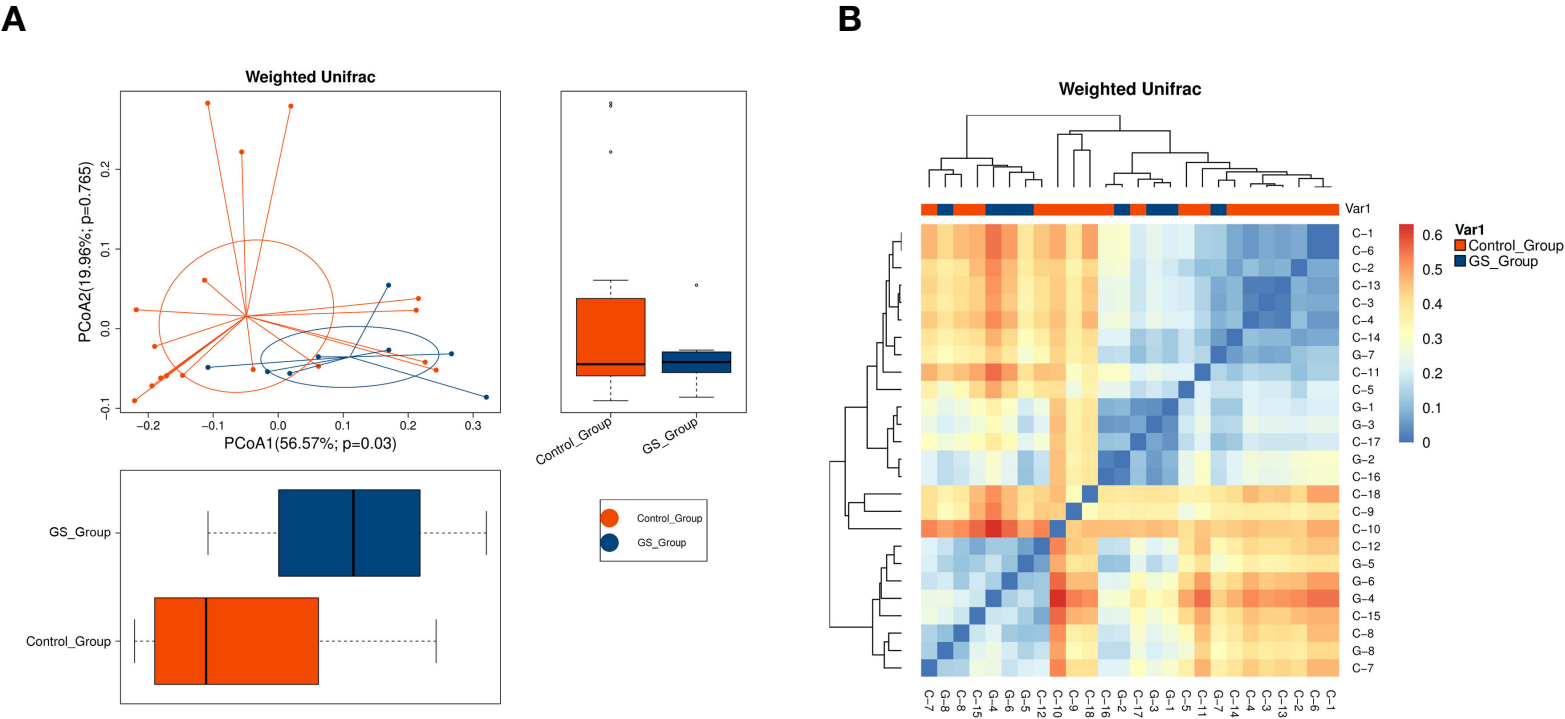
Distinct biliary microbial composition and distribution were identified in patients with giant CBDS by beta diversity analysis: **(A)** PCoA analysis using weighted UniFrac distance (*p*=0.030 on PCoA1 axis); **(B)** heatmap using weighted UniFrac distance (“G” for GS group, “C” for control group).

Richness of biliary microbial community was firstly compared. No significant difference was found according to alpha diversity analysis, including Chao1, observed species, Shannon and Simpson indexes (**Figures 1A-D**; **Supplementary Table 3**). To distinguish giant CBDS from normal CBDS, beta diversity analysis was further conducted. PCoA and NMDS analyses indicated the significant differences between the two groups (**Figure 2A**, **Supplementary Figure 2A**). And *p* value was calculated as 0.036 by Adonis test (**Supplementary Figure 2B**). The difference in microbial composition and distribution was further evident in the heatmap (**Figure 2B**). Therefore, the composition and distribution of biliary microbiota in patients with giant CBDS significantly differed from normal CBDS, even though they shared similar microbial richness.

### OTU and annotation analysis of biliary microbiota

**Figure 3:**
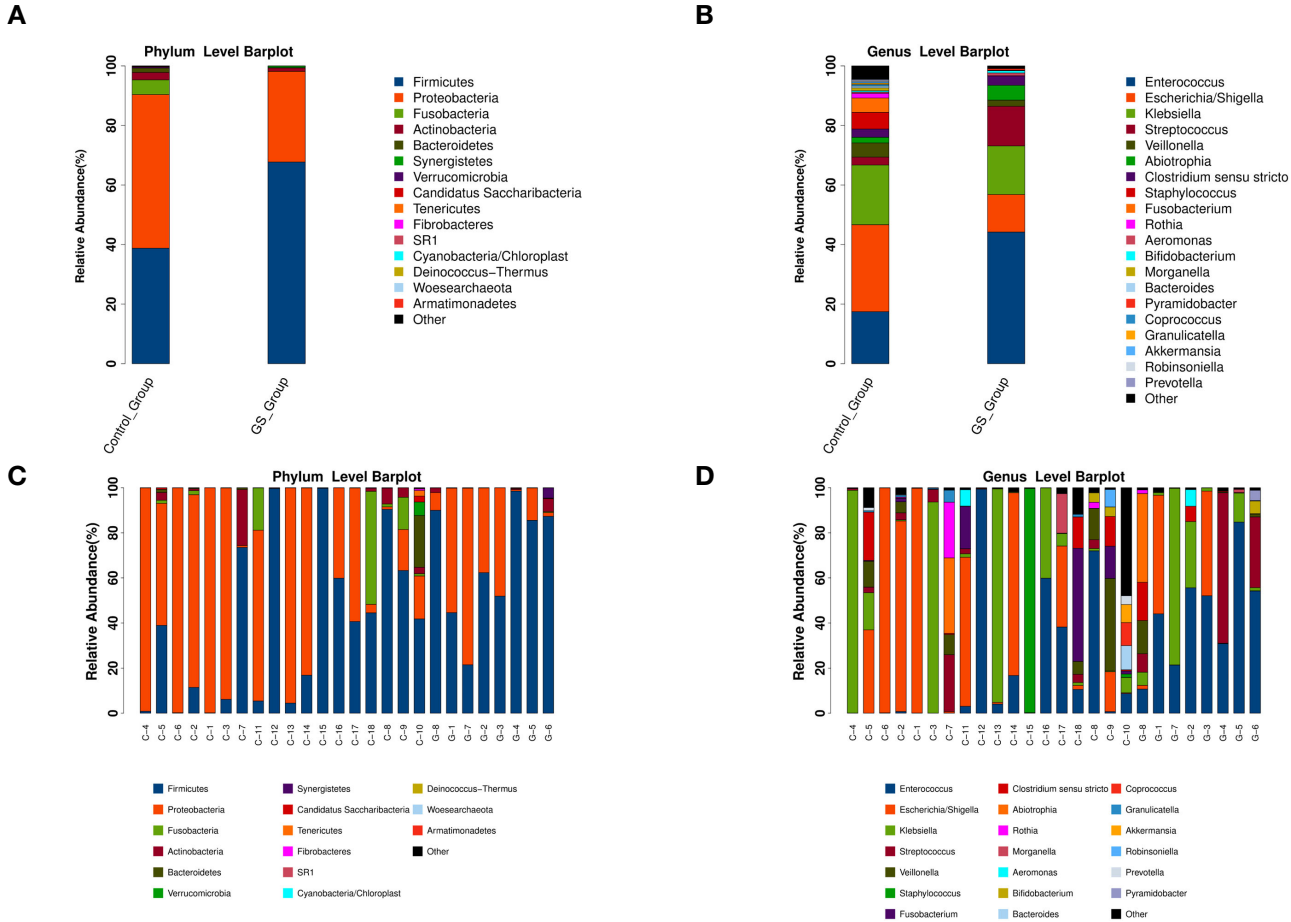
The relative abundance and distribution of biliary microbiota at different taxonomic levels (“G” for GS group, “C” for control group): **(A)** comparation at phylum level; **(B)** comparation at genus level; **(C)** microbial community at phylum level in each sample; **(D)** microbial community at genus level in each sample.

For acquiring detailed information of biliary microbiota, the relative abundance of detected bacteria was calculated and compared. The numbers of OTUs assignment to different taxonomic levels were showed on **Supplementary Table 4**. Firmicutes and Proteobacteria were the major phyla among all the individuals (**Figure 3C**), and Enterococcus, Escherichia/Shigella and Klebsiella were the dominant genera (**Figure 3D**). But the composition of these bacteria exhibited marked variations at each taxonomic level (**Figures 3A, B**, **Supplementary Figure 3**).

In detail, Proteobacteria accounted for 51.57% at phylum level in control group, followed by Firmicutes (38.74%), Fusobacteria (4.93%), Actinobacteria (2.50%) and Bacteroidetes (1.42%). On the contrary, Firmicutes (67.68%) played a dominant role in GS group, rather than Proteobacteria (30.42%), Actinobacteria (1.11%), Synergistetes (0.57%) or Bacteroidetes (0.09%). At genus level, the top five genera in GS group were Enterococcus (44.0%), Klebsiella (16.23%), Streptococcus (12.27%), Escherichia/Shigella (12.53%) and Abiotrophia (4.93%), while Escherichia/Shigella (29.15%), Klebsiella (19.93%), Enterococcus (17.31%), Staphylococcus (5.59%) and other unclassified bacteria (5.48%) were the dominant genera in control group.

**Figure 4:**
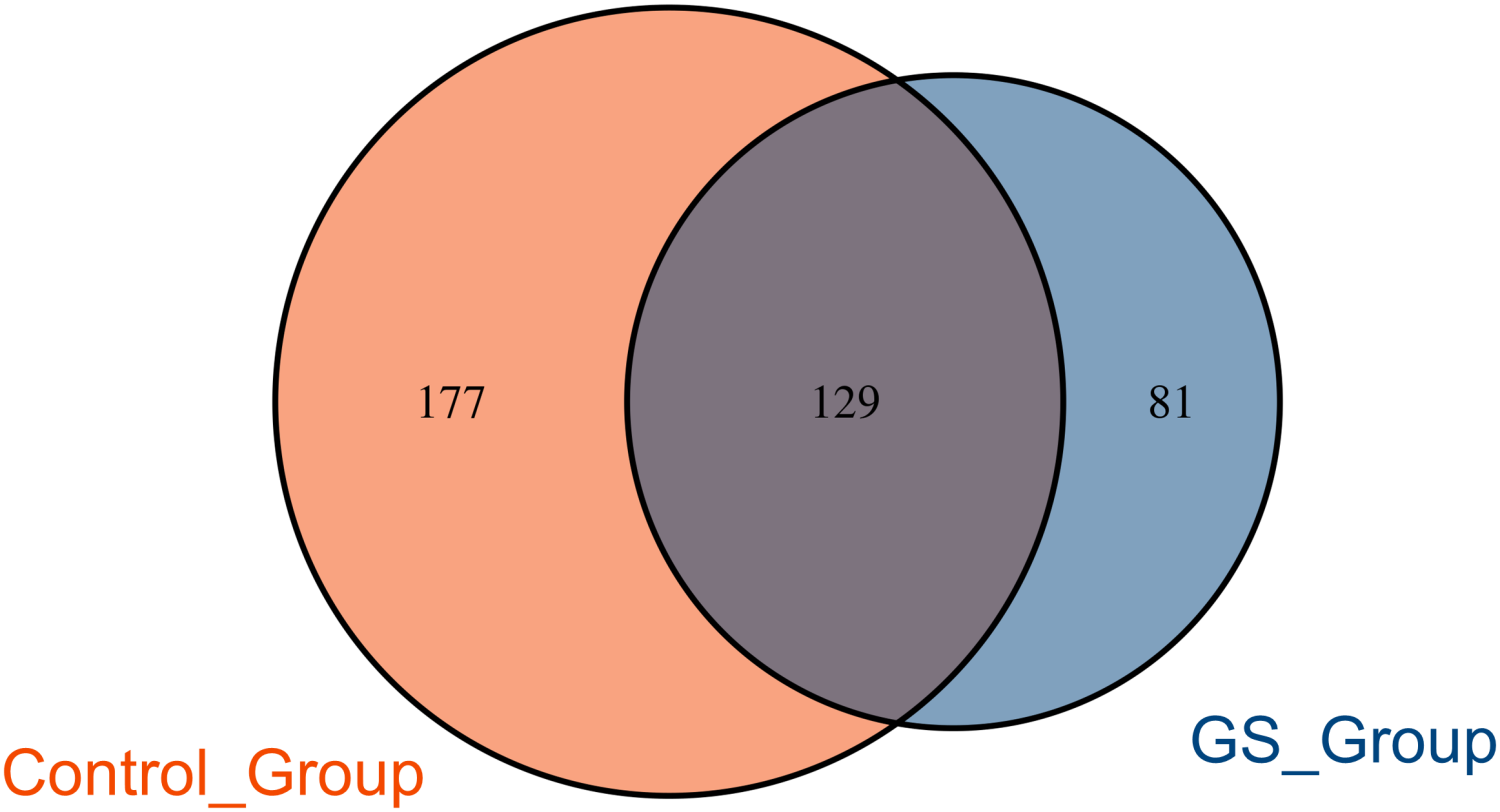
Venn diagram showed the unique and shared OTUs in GS and control groups.

There were 306 OTUs and 210 OTUs identified in control group and GS group, respectively, of which 129 OTUs were shared between the two groups (**Figure 4**). In this study, the OTU which was shared by all the individuals could be defined as the core microbiome (**Supplementary Figure 4**). As a result, the genus Klebsiella was the core microbiome.

### Characteristics of biliary microbiota in patients with giant CBDS

**Figure 5:**
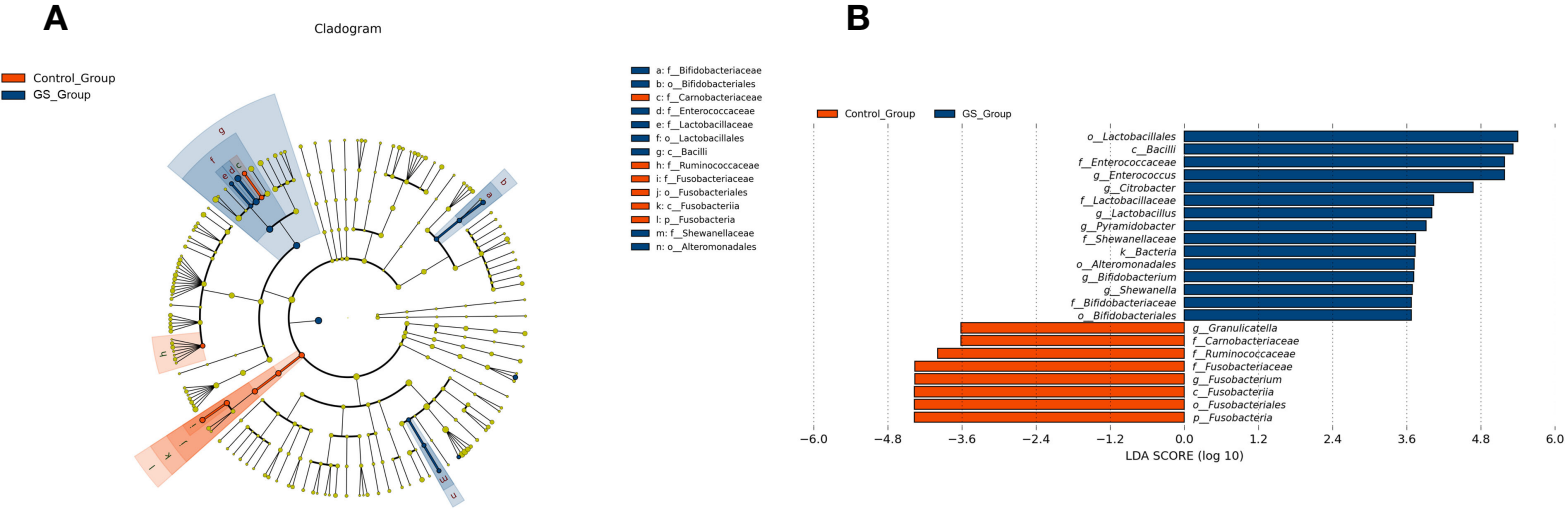
Characteristics of biliary microbiota in patients with giant CBDS: **(A)** LEfSe analysis revealed the different composition and the unique predominant bacteria; **(B)** the relative abundance of the 23 significantly different bacteria (LDA score threshold was 2.0).

We conducted further analyses to demonstrate the significant differences between the two groups. LEfSe analysis revealed the distinct composition of biliary microbiota and the unique predominant components in the two groups (**Figure 5A**). The relative abundance of 23 bacteria were significantly different (**Figure 5B**). 8 of the 23 bacteria were enriched in control group, while the other 15 bacteria were abundant in GS group. Enterococcus, Citrobacter, Lactobacillus, Pyramidobacter, Bifidobacterium and Shewanella were significantly abundant at genus level in GS group (**Figure 5B**). Moreover, Pyramidobacter was exclusively found in GS group according to annotation analysis, accompanied with the absence of Robinsoniella and Coprococcus (**Supplementary Table 5**).

Furthermore, spearman correlation analysis was performed for control group between patient age and genera abundance. Each genus whose relative abundance was in the top 20 was included into the analysis, except for Pyramidobacter (not existed in the control group) and unclassified genera. As **Supplementary Table 6** showed, neither positive correlation nor negative correlation between age and genera abundance could be found.

The genera with significant abundance differences between the two groups were revealed by Wilcoxon test as well (**Supplementary Table 7**). Similar to the aforementioned results, Enterococcus, Bifidobacterium, Lactobacillus, Pyramidobacter and Citrobacter were significantly abundant in GS group. And they contributed to the distinct biliary microbial composition (**Supplementary Figure 5**). The genera abundant in GS group showed obvious positive correlation with each other (**Supplementary Figure 6**). Meanwhile, Granulicatella and Fusobacterium were found abundant in control group (**Supplementary Table 7**).

### Changes of biliary metabolism

**Figure 6:**
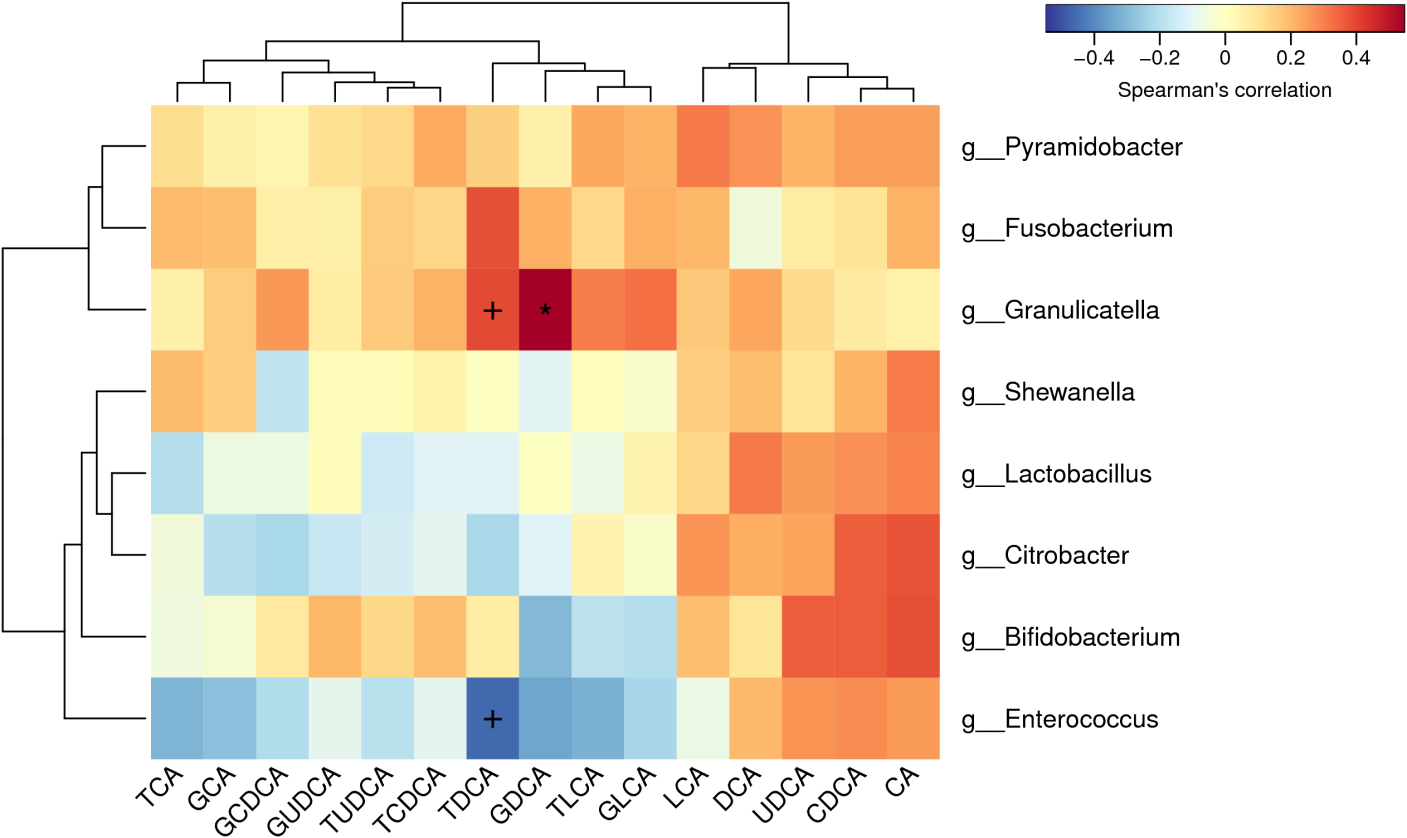
Correlation analysis between biliary microbiota and bile acids: Glycodesoxycholic acid (GDCA) had positive correlation with Granulicatella. Taurodeoxycholic acid (TDCA) had negative correlation with Enterococcus and positive correlation with Granulicatella.

Bile acids were successfully extracted from all the bile samples. According to the quantitative calculation, several free bile acids were significantly enriched in GS group (**Table 2**), including cholic acid (CA) (GS group *vs.* control group, 98.39μmol/mL *vs.* 26.15μmol/mL, *p*=0.035), chenodesoxycholic acid (CDCA) (GS group *vs.* control group, 54.69μmol/mL *vs.* 5.86μmol/mL, *p*=0.022) and ursodeoxycholic acid (UDCA) (GS group *vs.* control group, 2.70μmol/mL *vs.* 0.17μmol/mL, *p*=0.047). On the other hand, we observed an obvious decreasing tendency of some conjugated bile acids, such as glycocholic acid (GCA), glycodesoxycholic acid (GDCA), glycolithocholic acid (GLCA) and taurodeoxycholic acid (TDCA). In order to exclude the influence of age on bile acid metabolism, spearman correlation analysis was carried out again. There was no significant correlation between age and bile acids, either (**Supplementary Table 8**). Furthermore, correlation analysis between microbiota and bile acids indicated that TDCA had negative correlation with Enterococcus, however, positive correlation with Granulicatella (**Figure 6**).

**Table 2:** UPLC-MS/MS^†^ analysis of bile acids.

Bile acid^‡^	GS group n=8	Control group n=18	*p* value
CA	98.39 (6.18, 734.75)	26.15 (0.17, 1051.84)	0.035
DCA	4.35 (0.36, 88.71)	1.49 (0.08, 266.99)	0.264
CDCA	54.69 (11.37, 410.25)	5.86 (0.18, 2366.36)	0.022
UDCA	2.70 (0.48, 40.13)	0.17 (0.02, 1460.43)	0.047
LCA	0.32 (0.01, 5.90)	0.07 (0.01, 40.63)	0.350
GCA	5311.42 (2561.20, 9260.50)	7528.94 (400.25, 16094.48)	0.285
GDCA	281.42 (44.47, 2007.41)	543.34 (3.52, 7326.02)	0.057
GCDCA	5336.28 (3293.48, 7912.66)	5402.03 (167.72, 19409.06)	1.000
GUDCA	223.09 (23.22, 2002.61)	195.92 (4.63, 12365.51)	0.724
GLCA	3.18 (0.53, 78.86)	7.05 (0.04, 174.76)	0.495
TCA	1293.22 (896.14, 2570.70)	1579.26 (240.22, 4421.31)	0.495
TDCA	106.20 (8.80, 900.29)	326.59 (4.29, 1738.79)	0.144
TCDCA	1582.12 (708.62, 4977.37)	1620.18 (279.42, 7218.59)	0.892
TUDCA	38.16 (3.74, 526.23)	34.24 (1.85, 1479.22)	0.892
TLCA	1.64 (0.13, 19.93)	1.08 (0.07, 43.39)	0.807

**^†^** UPLC-MS/MS: ultra performance liquid chromatography-tandem mass spectrometry.

^‡^The concentration of bile acids is micromole per milliliter and described as median (minimum, maximum).

CA, cholic acid; DCA, deoxycholic acid; CDCA, chenodesoxycholic acid; UDCA, ursodeoxycholic acid; LCA, lithocholic acid; GCA, glycocholic acid; GDCA, glycodesoxycholic acid; GCDCA, glycochenodeoxycholic acid; GUDCA, glycoursodeoxycholic acid; GLCA, glycolithocholic acid; TCA, taurocholic acid; TDCA, taurodeoxycholic acid; TCDCA, taurochenodeoxycholic acid; TUDCA, tauroursodeoxycholic acid; TLCA, taurolithocholic acid.

**Figure 7:**
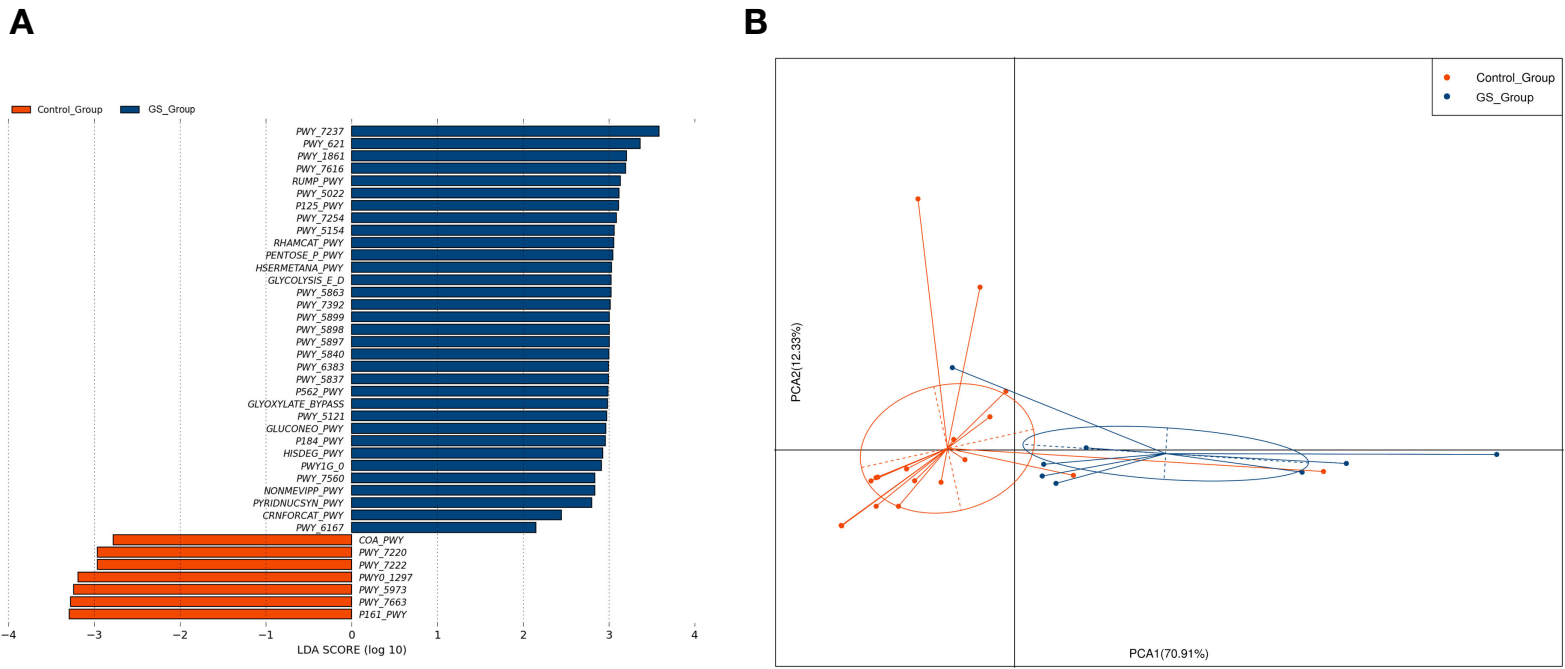
Metabolic pathways were calculated by PICRUSTs 2.0 based on the abundance of biliary microbiota to predict the metabolic functions: **(A)** 33 and 7 metabolic pathways were found enriched in GS group and control group, respectively; **(B)** PCA analysis indicated that metabolic functions of biliary microbiota in GS group differed from control group.

To explore the potential functions which might facilitate the formation of giant CBDS, we performed PICRUSt 2.0 calculation based on the abundance of biliary microbiota. There were 33 and 7 metabolic pathways enriched in GS group and control group, respectively (**Figure 7A**). Metabolic functions of biliary microbiota in GS group showed obvious differences from control group (**Figure 7B**).

## Discussion

ERCP has emerged as the primary choice for managing CBDS owing to its exceptionally minimal invasiveness and rapid post-procedural recovery (Williams et al., 2017; Committee et al., 2019; Manes et al., 2019). More than 90 percent of patients with ordinary CBDS can be successfully treated by ERCP (Okuno et al., 2016). Technical success rate approaches to 95-97% under experienced endoscopists (Tantau et al., 2013). However, numerous of stones, unusual shape, incarcerated location and large diameter restrict the success of ERCP, of which giant CBDS play a vital role (Williams et al., 2017; Committee et al., 2019; Manes et al., 2019). Approximately 10-15 percent of CBDS are too large to be removed by standard ERCP (Doshi et al., 2018). No accepted definition about giant CBDS has been widely used until now (Horiuchi et al., 2010; Fan et al., 2011; Hong et al., 2011). The lower limit was defined ranging from 12mm to 20mm. According to the latest European guideline (Manes et al., 2019), CBDS with a diameter lager than 15mm usually increase the difficulty of stone extraction. For another thing, relationship between stone and CBD lower segment influences the feasibility of stone removal (Sharma and Jain, 2008). Therefore, we set the two criteria for giant CBDS in this study, including stone diameter and the diameter ratio of stone against CBD, in order to make the study design more comprehensive and careful.

Biliary tract was recognized as a sterile environment for a long time, leading to the initial exclusion of microorganisms as contributors to the formation of cholelithiasis (Nicoletti et al., 2020). Benefit from the development of next generation sequencing and bioinformatic analysis, it has become increasingly convenient and feasible to conduct research about microbiota. From Maki. *et, al.* (Maki, 1966), several researchers have found the evidence of microbial colonization in biliary system by a series of methods (Nicoletti et al., 2020), including bacterial culture, scanning electronic microscopy, polymerase chain reaction and high-throughput sequencing. Wu. *et, al.* (Wu et al., 2013) employed 16S rRNA sequencing to compare the microbiota among fecal, bile and gallstones. Relative abundance and diversity of microbiota in bile were significantly different from gut. But the stones shared 85 percent of OTUs with bile. Molinero. *et, al.* (Molinero et al., 2019) used liver transplant donors as control to acquire nature bile. It was innovatively designed to prove the existence of biliary microbiota in healthy population. Firmicutes, Bacteroidetes, Proteobacteria and Actinobacteria were dominant phyla in biliary microbial niches, either non-diseases or with cholelithiasis.

A series of studies revealed the potential role of biliary microbiota in different diseases, including hepatobiliary tumor (Chen et al., 2019; Song et al., 2020), primary sclerosing cholangitis (Pereira et al., 2017), primary biliary cirrhosis (Hiramatsu et al., 2000) and recurrent choledocholithiasis (Tan et al., 2022). There existed a special microbial cluster in bile of all the individuals, different from gut flora. The composition of biliary microbiota varied from disease to disease. The relative abundance of core microbiome potentially influenced biological and medical characteristics of disorders. These unique microbial niches may offer valuable insights for the differential diagnosis and treatment of hepatobiliary diseases.

To the best of our knowledge, no study has focused on the characteristics of biliary microbiota in patients with giant CBDS. Like the previous studies (Wu et al., 2013; Molinero et al., 2019), Firmicutes and Proteobacteria governed the biliary microenvironment in both the two groups, rather than Bacteroidetes, the predominant phylum in gut. Even though the richness of biliary microbiota was comparable between GS group and control group, the microbial composition differed from each other significantly according to beta diversity. Firmicutes and Proteobacteria dominated GS group and control group, respectively. According to the results in **Supplementary Table 6**, the changes in 16S rRNA observed were not affected by the age of the donors.

The amount of OTUs decreased in GS group. A shift from gram-negative bacteria in control group to gram-positive bacteria in GS group was observed. Enterococcus accounted for less than 20 percent of the biliary microbial genera in control group, however, markedly increased to 44 percent in GS group. In the present study, Klebsiella was demonstrated as the core microbiome at genus level among all the patients. According to Kose. *et, al.* (Kose et al., 2018), Klebsiella was of bile resistance and involved in biofilm formation in gallstones. The unique role of Klebsiella in cholangiolithiasis was worthy of further study.

It deserves to be mentioned that the one-man show of Pyramidobacter in GS group. Pyramidobacter was proved to have bile resistance as well (Ye et al., 2016). It remains to be testified the potential function of Pyramidobacter on volume enhancement of CBDS by further experiments. Not only some single microorganism contributed to the formation of giant CBDS, but also the interaction among the abundant genera played an important role. It is meaningful to use the significant difference of microbial amount and composition to predict the risk for giant CBDS.

Cholestasis is considered to contribute to the formation of CBDS (Petrescu and DeMorrow, 2021). Accumulation of hydrophobic bile acids in the biliary system increases the risk of cholestasis. Jia. *et, al.* (Jia et al., 2020) demonstrated that the inhibition of geranylgeranyl diphosphate synthase (GGPPS) could ameliorate cholestasis by decreasing hydrophobic bile acids. Free bile acids are generally more hydrophobic than conjugated acids, such as CA and CDCA in the present study. The superpathway of geranylgeranyl diphosphate biosynthesis (PWY-5121) was enriched in GS group at the same time. Taking the decrease of conjugated bile acids into consideration, there was an increased risk of CBDS formation in GS group. Several metabolic pathways concerning cholelithiasis (Shen et al., 2015; Han et al., 2021) were also found abundant in GS group, including gluconeogenesis (GLUCONEO-PWY), glycolysis (GLYCOLYSIS-E-D) and L-methionine biosynthesis (HSERMETANA-PWY). Therefore, the special niches of biliary microbiota in GS group altered the metabolism in bile, resulting in the formation of giant CBDS. Further experiments are pregnant to verify the pathogenesis.

Compared with previous studies focusing on fecal microbiota, the sample size remains relatively small. A large number of patients were initially admitted to other hospitals due to cholangitis, where they received antibiotic therapy to alleviate symptoms. The patients were transferred to our hospital for ERCP when they recovered from acute symptoms. This study was singly conducted in our hospital prospectively. We could not control the antibiotics application. As a result, these patients did not meet the inclusion criteria. For another thing, numerous patients failed to meet the specific definition of giant CBDS. But the standards employed in this study has given an overall consideration to giant CBDS, including diameter of stone and bile duct. It was more comprehensive and careful to perform the research on giant CBDS. Multiple center studies and more convenient referral process are necessary to verify the conclusions in the future.

To sum up, the present study offered new insights into the characteristics of biliary microbiota in patients with giant CBDS. Distinct microbial composition is available to assess the disorders. Meanwhile, the metabolic functions which are relevant to cholelithiasis were abundant in biliary microbiota of giant CBDS. The findings of this study innovatively highlighted the importance of biliary microbiota in the etiology of giant CBDS.

## Data availability statement

16S rRNA sequencing data are available from the National Center for Biotechnology Information BioProject database under accession no. PRJNA979335.

## Ethics statement

The studies involving humans were approved by ethics committee of First Affiliated Hospital of Soochow University. The studies were conducted in accordance with the local legislation and institutional requirements. The participants provided their written informed consent to participate in this study. Written informed consent was obtained from the individual(s) for the publication of any potentially identifiable images or data included in this article.

## Author contributions

CD: Conceptualization, Data curation, Funding acquisition, Investigation, Methodology, Writing – original draft. CX: Funding acquisition, Methodology, Writing – original draft. LZ: Methodology, Software, Writing – original draft. MW: Data curation, Funding acquisition, Software, Writing – original draft. ZF: Conceptualization, Supervision, Writing – review & editing. JY: Conceptualization, Investigation, Methodology, Supervision, Writing – review & editing. DS: Conceptualization, Methodology, Supervision, Writing – review & editing.

## Supplementary material

Supplementary Figure 1 Sequencing was at the enough depth: **(A)** average sequence length was between 420bp and 440bp; **(B)** goods coverage index was comparable between the two groups (*p*=0.230).

Supplementary Figure 2 Composition of biliary microbiota was significantly different between the two groups: **(A)** NMDS analysis; **(B)** Adonis test using weighted UniFrac distance (*p*=0.036).

Supplementary Figure 3 The relative abundance and distribution of biliary microbiota at different taxonomic levels (“G” for GS group, “C” for control group): **(A)** comparation at class level; **(B)** comparation at order level; **(C)** comparation at family level; **(D)** microbial community at class level in each sample; **(E)** microbial community at order level in each sample; **(F)** microbial community at family level in each sample.

Supplementary Figure 4 Core microbiome was defined as the OTU which was shared by all the individuals.

Supplementary Figure 5 PCA analysis based on the Wilcoxon test indicated that the distribution of biliary microbiota differed markedly between the two groups.

Supplementary Figure 6 Spearman correlation analysis showed the positive correlation with each other among the genera abundant in GS group.
