# Supplementary figures and images for: Characteristics and metabolic potential of biliary microbiota in patients with giant common bile duct stones
Source: Front Cell Infect Microbiol. 2023 Nov 6;13:1259761. doi: 10.3389/fcimb.2023.1259761 (PMC10661410; doi:10.3389/fcimb.2023.1259761)

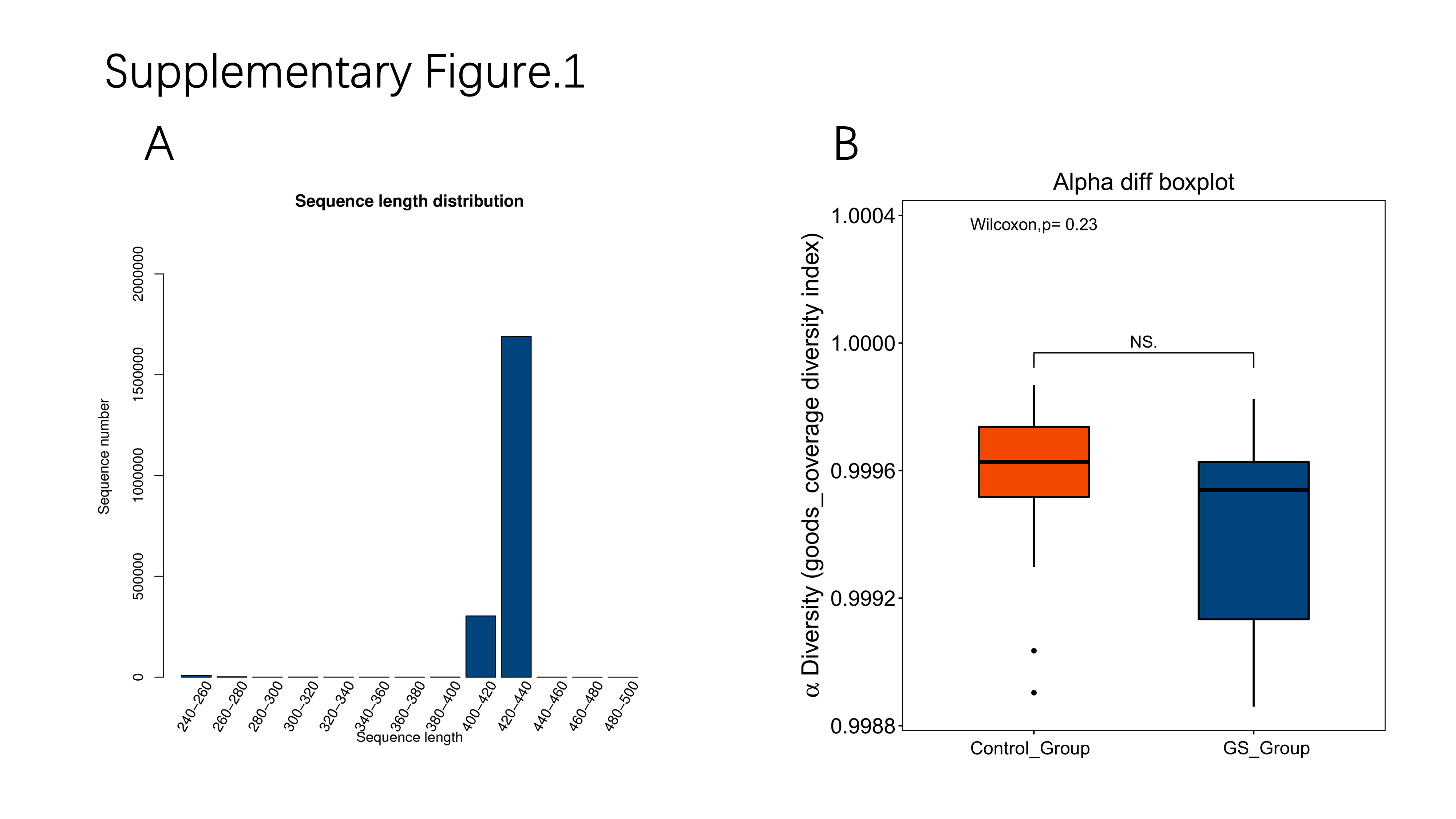

Supplement: Supplementary Figure 1 — Sequencing was at the enough depth: (A) average sequence length was between 420bp and 440bp; (B) goods coverage index was comparable between the two groups (p=0.230). [file Image_1.tif]

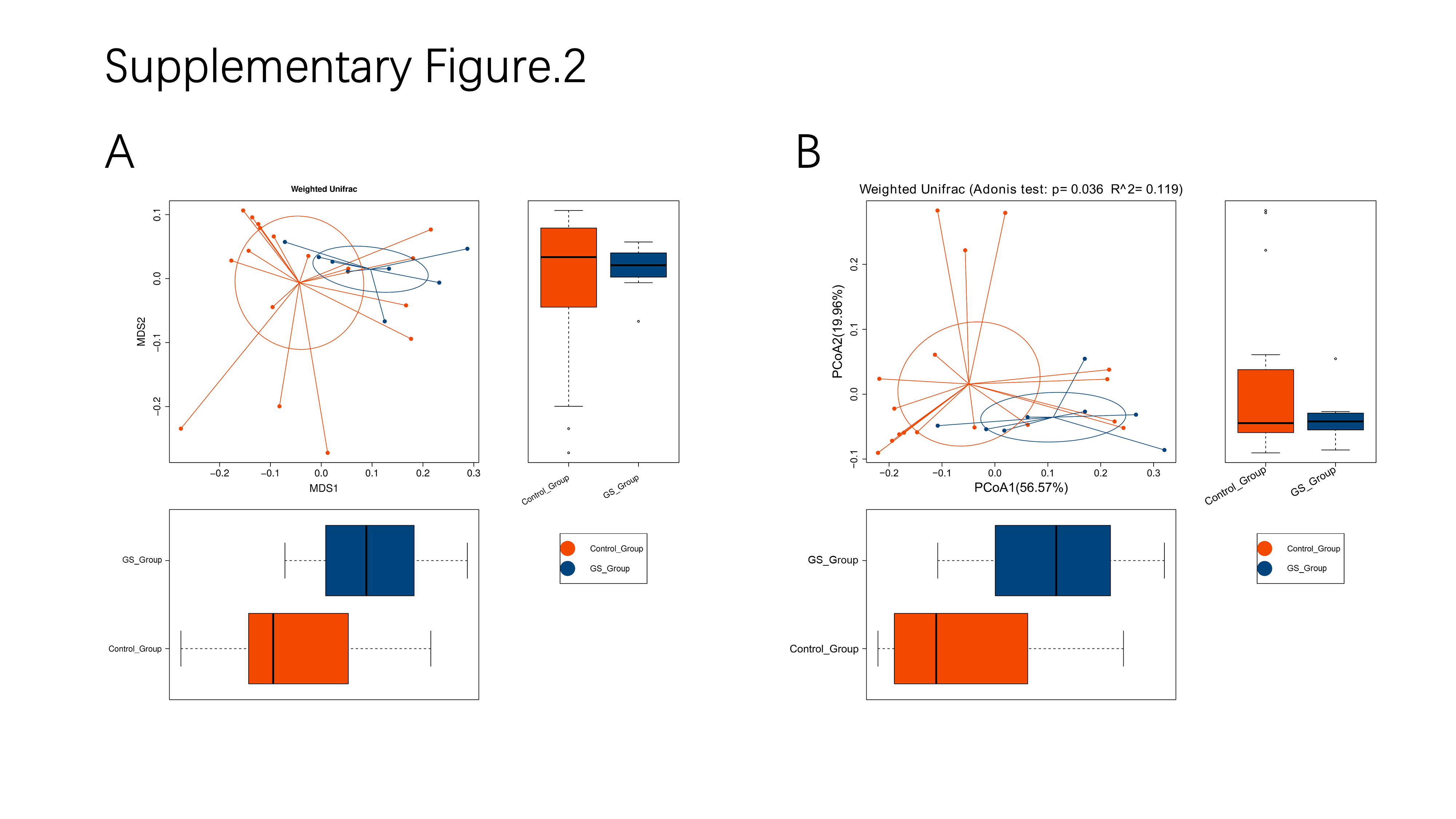

Supplement: Supplementary Figure 2 — Composition of biliary microbiota was significantly different between the two groups: (A) NMDS analysis; (B) Adonis test using weighted UniFrac distance (p=0.036). [file Image_2.tif]

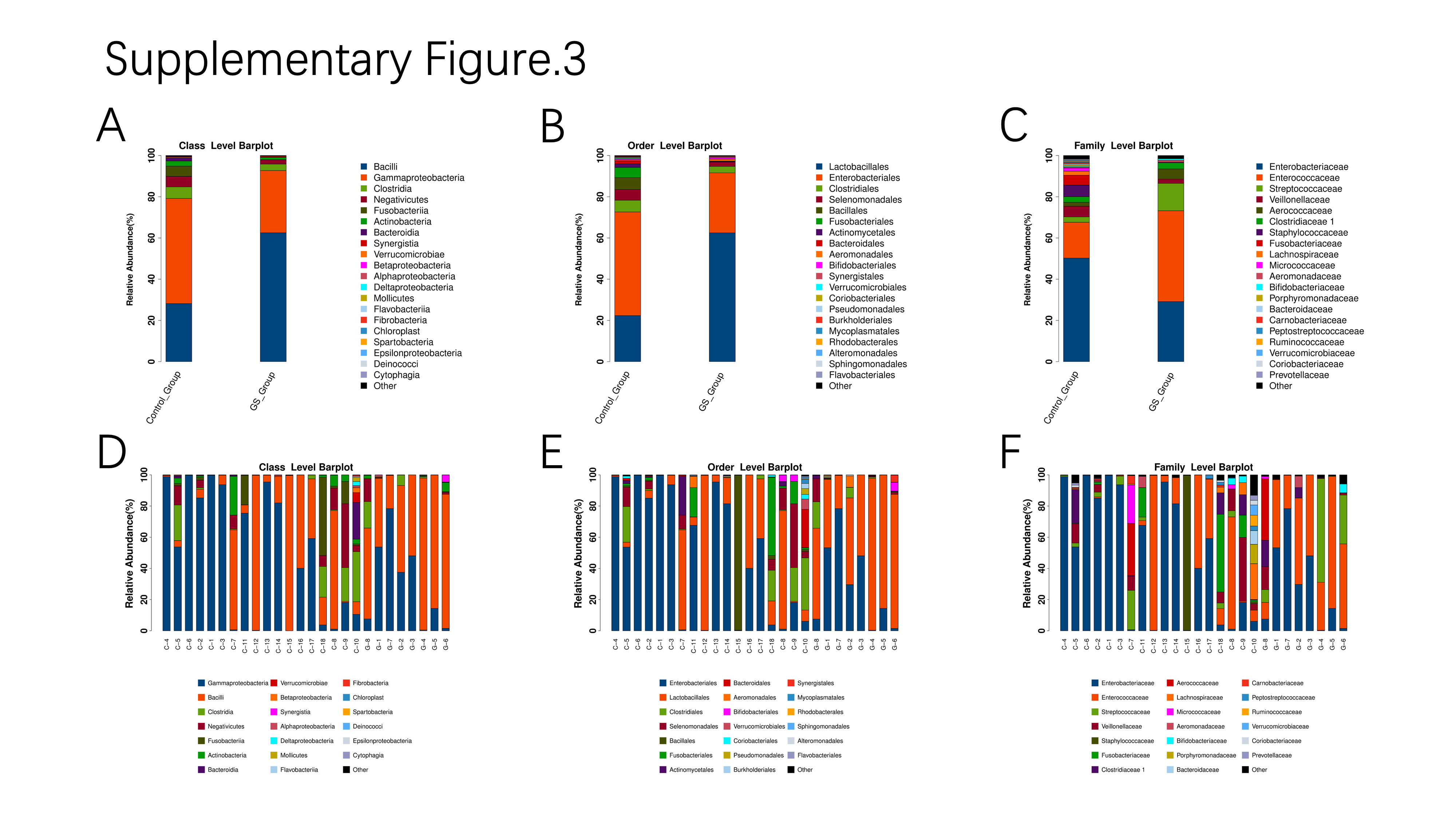

Supplement: Supplementary Figure 3 — The relative abundance and distribution of biliary microbiota at different taxonomic levels (“G” for GS group, “C” for control group): (A) comparation at class level; (B) comparation at order level; (C) comparation at family level; (D) microbial community at class level in each sample; (E) microbial community at order level in each sample; (F) microbial community at family level in each sample. [file Image_3.tif]

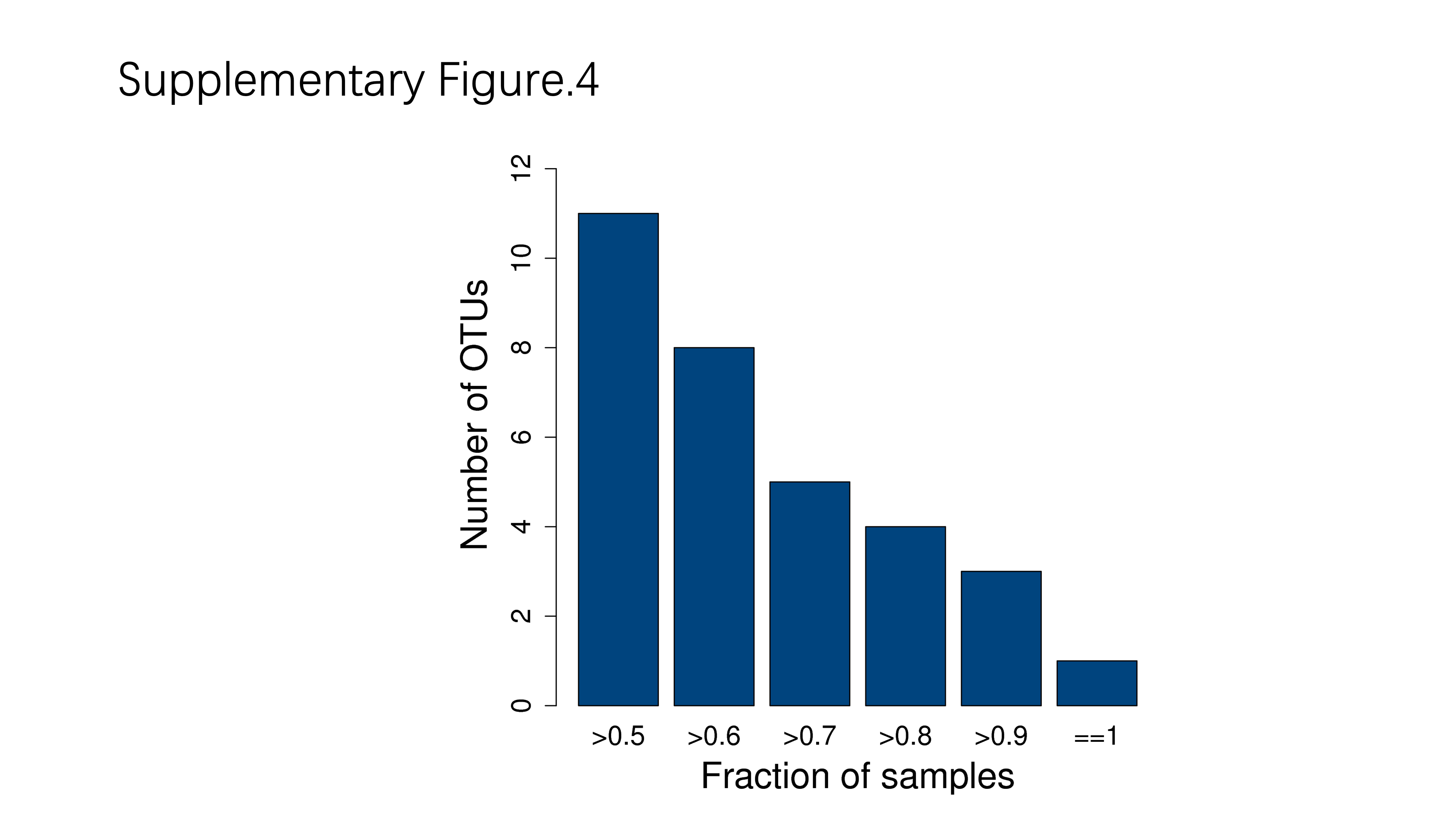

Supplement: Supplementary Figure 4 — Core microbiome was defined as the OTU which was shared by all the individuals. [file Image_4.tif]

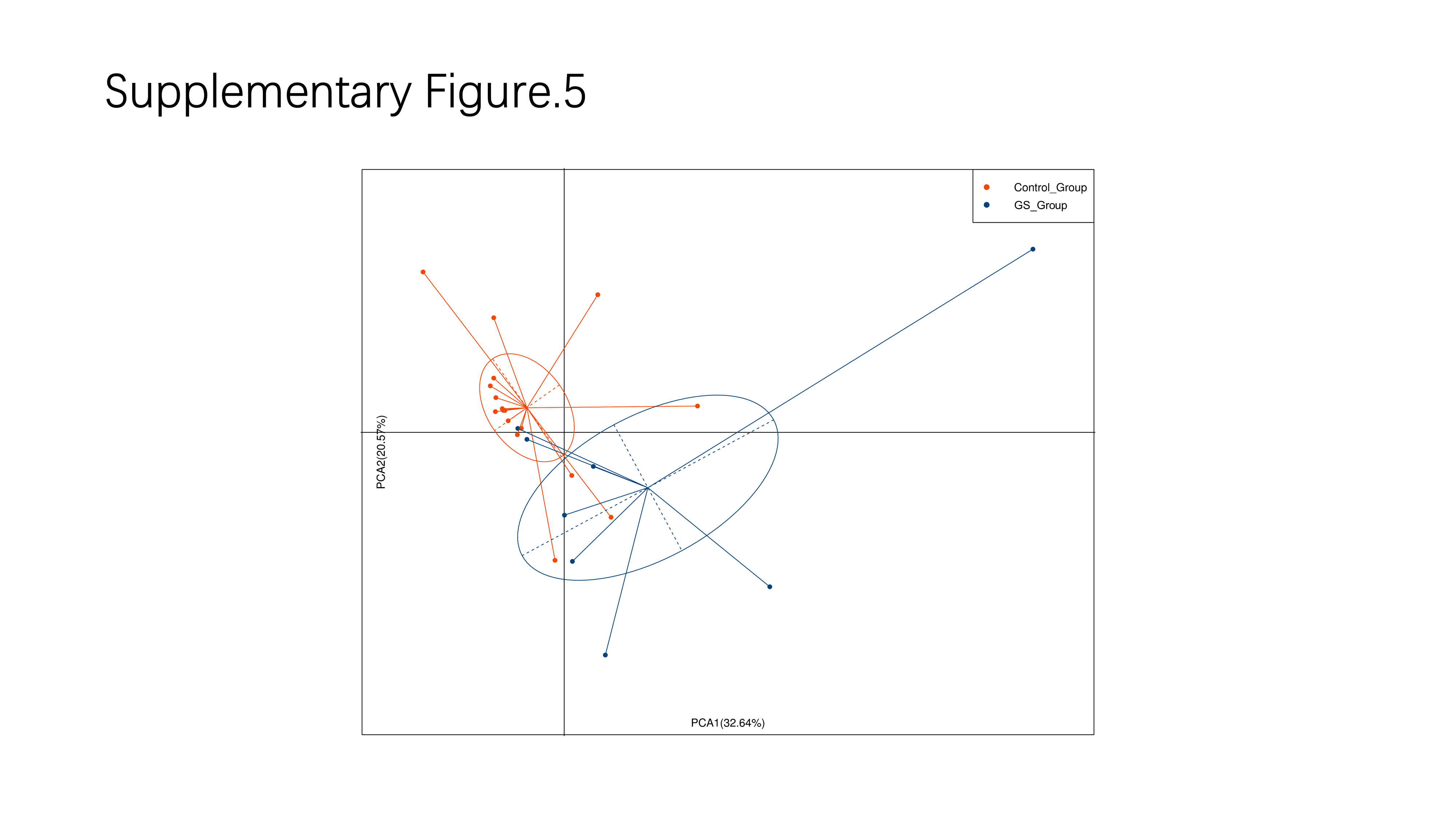

Supplement: Supplementary Figure 5 — PCA analysis based on the Wilcoxon test indicated that the distribution of biliary microbiota differed markedly between the two groups. [file Image_5.tif]

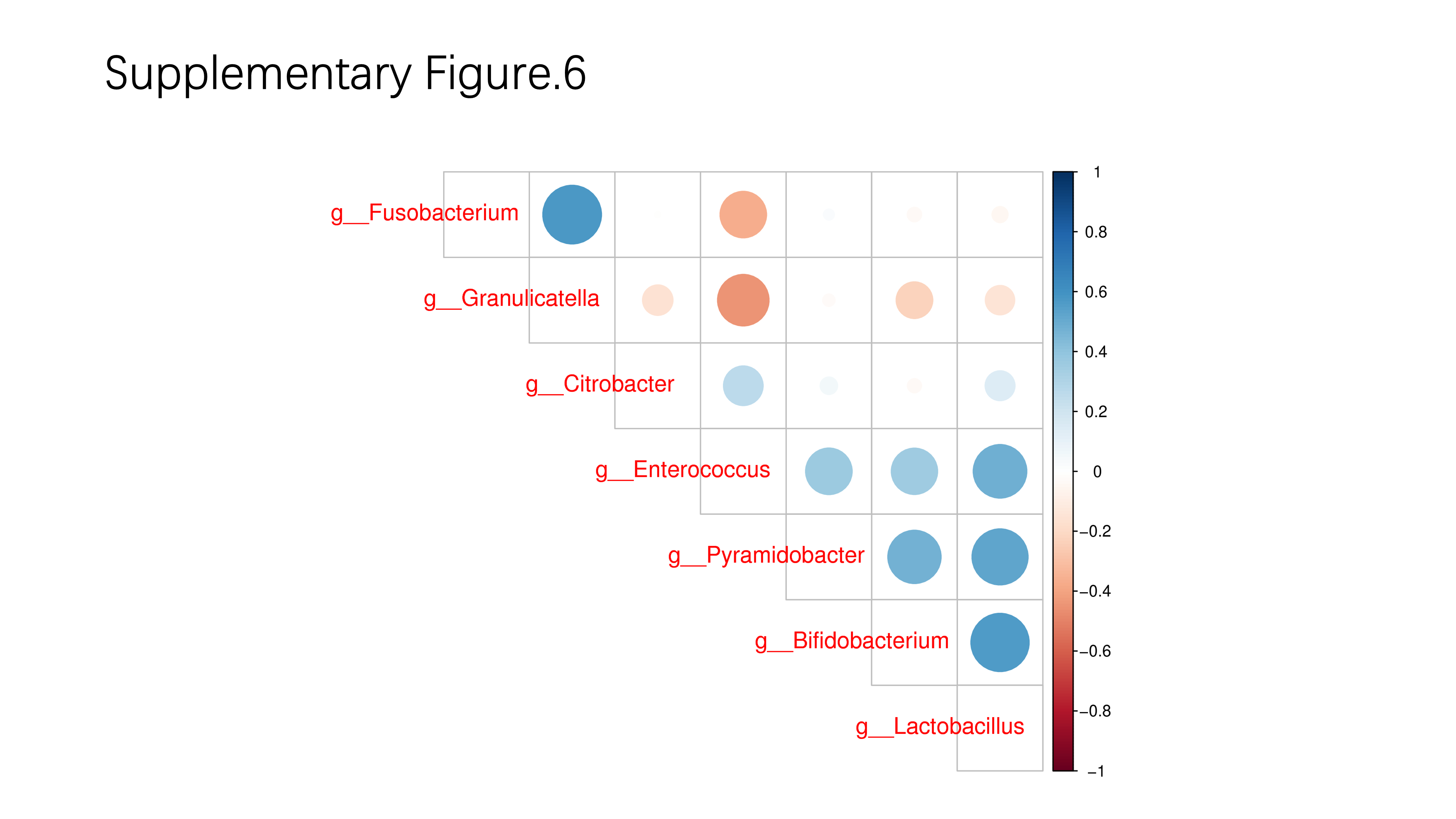

Supplement: Supplementary Figure 6 — Spearman correlation analysis showed the positive correlation with each other among the genera abundant in GS group. [file Image_6.tif]
