# Supplementary material for: Characteristics and metabolic potential of biliary microbiota in patients with giant common bile duct stones
Source: Front Cell Infect Microbiol. 2023 Nov 6;13:1259761. doi: 10.3389/fcimb.2023.1259761 (PMC10661410; doi:10.3389/fcimb.2023.1259761)
Supplement: Supplementary file 9 [file Table_3.docx]

**Supplementary Table.3 Alpha diversity analysis of biliary microbiota**

| Index | GS group  n=8 | Control group  n=18 | *p* value |
| --- | --- | --- | --- |
| goods coverage (mean) | 1.00 | 1.00 | 0.230 |
| Chao1 (mean) | 56.73 | 50.63 | 0.495 |
| observed species (mean) | 45.88 | 39.83 | 0.266 |
| Shannon (mean) | 1.61 | 1.46 | 0.531 |
| Simpson (mean) | 0.54 | 0.40 | 0.367 |

**Supplementary Table.4 Assignment of OTUs to different taxonomic levels**

| OTU name | Number |
| --- | --- |
| Assigned to Kingdom | 385 |
| Assigned to Phylum | 370 |
| Assigned to Class | 356 |
| Assigned to Order | 349 |
| Assigned to Family | 332 |
| Assigned to Genus | 276 |

**Supplementary Table.5 Relative abundance of the top 20 genera**

| Genera name | GS group  n=8 | Control group  n=18 |
| --- | --- | --- |
| Enterococcus | 43.99712906 | 17.31117781 |
| Escherichia/Shigella | 12.52990561 | 29.15041493 |
| Klebsiella | 16.23330723 | 19.93006317 |
| Streptococcus | 13.27428335 | 2.636526648 |
| Veillonella | 2.072730436 | 4.77595565 |
| Staphylococcus | 0.00652486 | 5.587696531 |
| Fusobacterium | 0.012505981 | 4.764839223 |
| Clostridium sensu stricto | 3.012310235 | 2.745032648 |
| Abiotrophia | 4.928987777 | 1.883992827 |
| Rothia | 0.2185828 | 1.550983321 |
| Morganella | 0.171277568 | 0.968579176 |
| Aeromonas | 0.91674279 | 0.403332995 |
| Bifidobacterium | 0.706859803 | 0.467131623 |
| Granulicatella | 0.001087477 | 0.476798082 |
| Bacteroides | 0.012505981 | 0.436440617 |
| Robinsoniella | 0 | 0.429190772 |
| Coprococcus | 0 | 0.414691084 |
| Akkermansia | 0.00652486 | 0.32696797 |
| Prevotella | 0.030993084 | 0.260269404 |
| Pyramidobacter | 0.574187655 | 0 |
| Unclassified genera | 1.293553439 | 5.479915515 |

**Supplementary Table.6 Spearman correlation analysis between age and genera abundance**

|  | Spearman rho | 95% CI^†^  lower limit | 95% CI^†^  upper limit | *p* value |
| --- | --- | --- | --- | --- |
| age - Enterococcus | 0.273 | -0.236 | 0.665 | 0.273 |
| age - Escherichia/Shigella | 0.090 | -0.406 | 0.545 | 0.723 |
| age - Klebsiella | -0.284 | -0.671 | 0.225 | 0.253 |
| age - Streptococcus | 0.020 | -0.463 | 0.494 | 0.938 |
| age - Veillonella | 0.108 | -0.391 | 0.557 | 0.671 |
| age - Staphylococcus | 0.320 | -0.188 | 0.692 | 0.196 |
| age - Fusobacterium | -0.152 | -0.588 | 0.352 | 0.546 |
| age - Clostridium sensu stricto | -0.126 | -0.570 | 0.375 | 0.619 |
| age - Abiotrophia | 0.446 | -0.041 | 0.762 | 0.063 |
| age - Rothia | 0.004 | -0.476 | 0.481 | 0.988 |
| age - Morganella | -0.296 | -0.679 | 0.212 | 0.232 |
| age - Aeromonas | -0.406 | -0.741 | 0.090 | 0.095 |
| age - Bifidobacterium | -0.153 | -0.588 | 0.351 | 0.545 |
| age - Granulicatella | 0.003 | -0.476 | 0.481 | 0.990 |
| age - Bacteroides | 0.207 | -0.302 | 0.624 | 0.410 |
| age - Robinsoniella | -0.211 | -0.626 | 0.298 | 0.401 |
| age - Coprococcus | 0.234 | -0.275 | 0.641 | 0.350 |
| age - Akkermansia | 0.329 | -0.177 | 0.698 | 0.182 |
| age - Prevotella | 0.259 | -0.251 | 0.656 | 0.300 |

^†^CI: Confidence interval

**Supplementary Table.7 Wilcoxon test at genus level**

| Genera name | GS group  n=8 | Control group  n=18 | *p* value |
| --- | --- | --- | --- |
| Bifidobacterium | 0.007069 | 0.004671 | 0.048761 |
| Citrobacter | 0.000359 | 7.49*10^-5 | 0.036543 |
| Enterococcus | 0.439971 | 0.173112 | 0.011465 |
| Fusobacterium | 0.000125 | 0.047648 | 0.034347 |
| Granulicatella | 1.09*10^-5 | 0.004768 | 0.049269 |
| Lactobacillus | 0.001702 | 0.000106 | 0.036381 |
| Pyramidobacter | 0.005742 | 0 | 0.035445 |

**Supplementary Table.8 Spearman correlation analysis between age and bile acids**

|  | Spearman rho | 95% CI^†^  lower limit | 95% CI^†^  upper limit | *p* value |
| --- | --- | --- | --- | --- |
| age - CA | -0.262 | -0.658 | 0.248 | 0.294 |
| age - DCA | 0.410 | -0.123 | 0.760 | 0.115 |
| age - CDCA | -0.171 | -0.600 | 0.335 | 0.498 |
| age - UDCA | -0.157 | -0.591 | 0.347 | 0.533 |
| age - LCA | 0.277 | -0.269 | 0.688 | 0.300 |
| age - GCA | -0.112 | -0.560 | 0.388 | 0.659 |
| age - GDCA | -0.031 | -0.515 | 0.469 | 0.907 |
| age - GCDCA | -0.181 | -0.607 | 0.326 | 0.472 |
| age - GUDCA | -0.298 | -0.679 | 0.211 | 0.230 |
| age - GLCA | 0.068 | -0.424 | 0.529 | 0.788 |
| age - TCA | -0.327 | -0.696 | 0.180 | 0.186 |
| age - TDCA | -0.043 | -0.511 | 0.444 | 0.864 |
| age - TCDCA | -0.274 | -0.665 | 0.235 | 0.271 |
| age - TUDCA | -0.315 | -0.690 | 0.192 | 0.202 |
| age - TLCA | 0.102 | -0.395 | 0.554 | 0.686 |

^†^CI: Confidence interval

CA: cholic acid; DCA: deoxycholic acid; CDCA: chenodesoxycholic acid; UDCA: ursodeoxycholic acid; LCA: lithocholic acid; GCA: glycocholic acid; GDCA: glycodesoxycholic acid; GCDCA: glycochenodeoxycholic acid; GUDCA: glycoursodeoxycholic acid; GLCA: glycolithocholic acid; TCA: taurocholic acid; TDCA: taurodeoxycholic acid; TCDCA: taurochenodeoxycholic acid; TUDCA: tauroursodeoxycholic acid; TLCA: taurolithocholic acid.
